# Evaluating the risk of hypertension in residents in primary care in Shanghai, China with machine learning algorithms

**DOI:** 10.3389/fpubh.2022.984621

**Published:** 2022-10-04

**Authors:** Ning Chen, Feng Fan, Jinsong Geng, Yan Yang, Ya Gao, Hua Jin, Qiao Chu, Dehua Yu, Zhaoxin Wang, Jianwei Shi

**Affiliations:** ^1^School of Public Health, Shanghai Jiao Tong University School of Medicine, Shanghai, China; ^2^School of Medicine, Tongji University, Shanghai, China; ^3^School of Medicine, Nantong University, Nantong, China; ^4^School of Economics and Management, Tongji University, Shanghai, China; ^5^Department of General Practice, Yangpu Hospital, Tongji University School of Medicine, Shanghai, China; ^6^Shanghai General Practice and Community Health Development Research Center, Shanghai, China; ^7^Academic Department of General Practice, Tongji University School of Medicine, Shanghai, China; ^8^Clinical Research Center for General Practice, Tongji University, Shanghai, China; ^9^The First Affiliated Hospital of Hainan Medical University, Haikou, China; ^10^Department of Social Medicine and Health Management, School of Public Health, Shanghai Jiao Tong University School of Medicine, Shanghai, China; ^11^School of Management, Hainan Medical University, Haikou, China

**Keywords:** hypertension, risk assessment model, risk of hypertension, machine learning algorithms, primary care

## Abstract

**Objective:**

The prevention of hypertension in primary care requires an effective and suitable hypertension risk assessment model. The aim of this study was to develop and compare the performances of three machine learning algorithms in predicting the risk of hypertension for residents in primary care in Shanghai, China.

**Methods:**

A dataset of 40,261 subjects over the age of 35 years was extracted from Electronic Healthcare Records of 47 community health centers from 2017 to 2019 in the Pudong district of Shanghai. Embedded methods were applied for feature selection. Machine learning algorithms, XGBoost, random forest, and logistic regression analyses were adopted in the process of model construction. The performance of models was evaluated by calculating the area under the receiver operating characteristic curve, sensitivity, specificity, positive predictive value, negative predictive value, accuracy and F1-score.

**Results:**

The XGBoost model outperformed the other two models and achieved an AUC of 0.765 in the testing set. Twenty features were selected to construct the model, including age, diabetes status, urinary protein level, BMI, elderly health self-assessment, creatinine level, systolic blood pressure measured on the upper right arm, waist circumference, smoking status, low-density lipoprotein cholesterol level, high-density lipoprotein cholesterol level, frequency of drinking, glucose level, urea nitrogen level, total cholesterol level, diastolic blood pressure measured on the upper right arm, exercise frequency, time spent engaged in exercise, high salt consumption, and triglyceride level.

**Conclusions:**

XGBoost outperformed random forest and logistic regression in predicting the risk of hypertension in primary care. The integration of this risk assessment model into primary care facilities may improve the prevention and management of hypertension in residents.

## Introduction

Hypertension is becoming increasingly common in primary care. It is accompanied by the occurrence and development of a series of cardiovascular events, disability and even premature death if not detected early and managed well (1). An estimated 245 million adults are diagnosed with hypertension in China (2). An early warning after accurately evaluating the risk of hypertension in primary care patients can alert individuals in the healthy population or subhealthy population with unhealthy lifestyles to take measures to slow or stop the progression of hypertension. Similar practices have been implemented in foreign countries. For instance, management of risk factors for various chronic diseases has been implemented in primary care in Australia (3). Risk assessment models are a cost-effective measure for identifying high-risk individuals with chronic diseases (4, 5). Nevertheless, few existing models can be applied to the health management services provided in primary care. The most intractable problem is that most of these models are targeted at patients in a hospital setting (6); thus, the data input into the models are all extracted from the EHRs of hospitals, which may not be readily available in primary care settings and suitable for general practitioners to implement.

Machine learning (ML) is a nuclear branch of artificial intelligence that has been employed everywhere knowingly or unknowingly, not only in industry and the military but also in medicine and healthcare (7). As a modern data mining, extraction, and analysis technology, ML has the extraordinary ability to automatically train itself and improve its performance without human instruction or elaborate programming (8, 9). With the ability to identify a pattern or make a decision based on the knowledge input, ML algorithms have demonstrated their excellent performance in the area of risk evaluation of diseases. Higher accuracy separates ML algorithms from various other statistical methods. Highly precise risk prediction models for future hypertension were constructed using artificial intelligence techniques in Japan (10). Health check-up data from 18,258 Japanese individuals were utilized to develop a risk prediction model for new-onset hypertension by machine learning techniques. The XGBoost and ensemble models outperformed the logistic regression models [area under the receiver operating characteristic curve (AUC) = 0.859], with AUCs of 0.877 and 0.881, respectively. A study based on several easy-to-collect risk factors to predict the risk of hypertension also revealed that the random forest (AUC = 0.92), CatBoost (AUC = 0.87), and MLP neural network (AUC = 0.78) models performed better than the logistic regression analysis (AUC = 0.77) (11). Although ML is applicable in an extensive range of contexts, the ML algorithm technique alone is insufficient to solve real-world problems (12). Thus, health and medical data in a primary care setting were utilized to facilitate the practical implementation of the risk assessment model for residents in primary care.

The objective of this study is to develop and compare the performances of three ML algorithms on predicting the risk of hypertension for residents over the age of 35 years in primary care in Shanghai, China.

## Materials and methods

### Data source

The dataset was extracted from the electronic healthcare records of 47 community health centers in the Pudong district of Shanghai. Health records, health examinations and other health-related data of community residents over 35 years old from 2017 to 2019 were collected as the original set of data. A total of 40,261 subjects were enrolled in the study. The dataset included 20 variables containing information regarding demographic characteristics, diagnosis, biochemical indicators and lifestyles. The characteristics of the participants in primary care are shown in Table 1.

**Table 1:** Characteristics of the participants in primary care settings.

**Feature**	**Hypertension (*n* = 25,038)**	**Normal (*n* = 15,223)**	**χ^2^**	***P***
Age^*^	72.00 (68.00–78.00)	70.00 (66.00–75.00)	683.51^a^	<0.01
Diabetes status			2077.18^b^	<0.01
No	16,512 (65.95)	13,177 (86.56)		
Yes	8,526 (34.05)	2,046 (13.44)		
Urinary protein level			32.33^b^	<0.01
Negative	8,261 (32.99)	8,392 (55.13)		
Positive	581 (2.32)	405 (2.66)		
BMI^*^	24.98 (23.01–27.30)	24.16 (22.10–26.30)	458.44^a^	<0.01
EHSA			563.15^b^	<0.01
1	6,973 (27.85)	5,973 (39.24)		
2	12,604 (50.34)	6,387 (41.96)		
3	358 (1.43)	219 (1.44)		
4	277 (1.11)	149 (0.98)		
5	163 (0.65)	46 (0.30)		
Cr level^*^	69.00 (58.00–84.00)	66.00 (56.00–77.70)	229.09^a^	<0.01
SBP^*^	140.00 (130.00–153.00)	139.00 (126.00–148.00)	326.93^a^	<0.01
WC^*^	87.00 (81.00–93.00)	85.00 (79.00–91.00)	157.52^a^	<0.01
Smoking status			200.85^b^	<0.01
1	19,171 (76.57)	10,238 (67.25)		
2	1,159 (4.63)	857 (5.63)		
3	2,028 (8.10)	1,700 (11.17)		
LDL-C level^*^	2.89 (2.20–3.41)	2.99 (2.46–3.63)	402.35^a^	<0.01
HDL-C level^*^	1.35 (1.11–1.54)	1.40 (1.20–1.66)	586.65^a^	<0.01
Frequency of drinking			97.64^b^	<0.01
1	18,096 (72.27)	9,837 (64.62)		
2	2,753 (11.00)	1,771 (11.63)		
3	199 (0.79)	151 (0.99)		
4	918 (3.67)	764 (5.02)		
Glucose level^*^	5.60 (5.13–6.90)	5.50 (5.00–6.33)	247.31^a^	<0.01
Urea nitrogen level^*^	5.63 (4.80–6.83)	5.63 (4.80–6.37)	306.45^a^	<0.01
TC level^*^	4.82 (4.01–5.52)	4.99 (4.35–5.72)	267.34^a^	<0.01
DPB^*^	78.00 (72.00–84.00)	78.00 (70.00–82.00)	235.77^a^	<0.01
Exercise frequency			17.48^b^	<0.01
1	14,751 (58.91)	8,460 (55.57)		
2	815 (3.26)	391 (2.57)		
3	1,495 (5.97)	926 (6.08)		
4	5,471 (21.85)	3,331 (21.88)		
High salt consumption			17.24^b^	<0.01
No	24,938 (99.60)	15,199 (99.80)		
Yes	100 (0.40)	24 (0.20)		
TG level^*^	1.39 (1.12–1.84)	1.39 (1.00–1.80)	13.22^a^	<0.01
Time spent engaged in exercise^*^	30.00 (30.00–30.00)	30.00 (30.00–30.00)	0.41^a^	0.52

* Refers to nonnormally distributed measurement data, reported as the median (25th percentile, 75th percentile).

a refers to results of the rank sum test.

b refers to the results of the chi-square test.

### Definition of hypertension

Hypertension was defined as (1) systolic blood pressure (SBP) ≥140 mmHg and/or diastolic blood pressure (DBP) ≥ 90 mmHg, which was measured three times on different days in the clinic without the use of antihypertensive drugs, according to Chinese guidelines for the prevention and treatment of hypertension (2018 revised edition) (13) and/or (2) a diagnosis of hypertension by a physician and/or (3) antihypertension treatment.

### Inclusion and exclusion criteria

The sample data that fulfilled the following inclusion criteria were obtained for further analysis in this study: community residents over 35 years of age. The chapter “Health Management Service Specifications for Hypertension Patients” in “National Basic Public Health Service Specifications (the Third Edition)” specified that one of the services is to “Provide free blood pressure measurement once a year for permanent residents aged 35 years old and over in area of responsibility” (14). Therefore, we chose community residents aged 35 years and older as our subjects. The exclusion criteria were: (1) individuals who were unable to provide informed consent, (2) those have any diagnosis of secondary or gestational hypertension, and (3) those who could not cooperate with the investigation because of a long-term outing or a lack of electronic healthcare records.

### Data processing

Outliers were handled by interquartile range (IQR). The IQR is evaluated as IQR = Q3–Q1. Q3 is the upper quartile, and Q1 is the lower quartile. Outliers were defined as records that fell below Q1–(1.5^*^IQR) or above Q3+ (1.5^*^IQR).

Missing values, such as data with null rows and columns, which did not have a single value or number available, were deleted. Different methods, such as the mean values, median values, mode values, feature combinations and null values, were adopted for dealing with the individual missing values according to the characteristics of different variables. In total, 5.62% of missing values were found in the whole dataset.

Discretization was performed by splitting the range of the continuous variables into intervals to save time needed to build the risk assessment model and improve the assessment results (15).

### Feature selection

Feature selection, which is one of the essential parts of building a good prediction model, was employed in this study to improve the prediction accuracy by choosing the most important variables. Moreover, it facilitates a reduction in the resources (time and space) needed to construct the model (16). The embedded method was applied in this study for feature selection. It integrates the feature selection process with the model training process. This method considers variable interactions and is less computationally demanding than the wrapper method (17).

Twenty features were selected to construct the model, from the 127 variables (see the Supplementary Files): age, diabetes status, urinary protein level, BMI, elderly health self-assessment (EHSA), creatinine (Cr) level, systolic blood pressure measured on the upper right arm (SBP), waist circumference (WC), smoking status, low-density lipoprotein cholesterol (LDL-C) level, high-density lipoprotein cholesterol (HDL-C) level, frequency of drinking, glucose level, urea nitrogen level, total cholesterol (TC) level, diastolic blood pressure of the upper right arm (DBP), exercise frequency, time spent engaged in exercise, high salt consumption, and triglyceride (TG) level.

### Machine learning algorithms

Extreme Gradient Boosting (XGBoost) is a supervised ML algorithm (18). It is a scalable end-to-end tree boosting system (19). XGBoost can automatically perform parallel computations and is generally more than 10 times faster than GBM (20). Its input types include dense matrix, sparse matrix, data file and xgb.dmatrix. XGBoost accepts sparse input for both tree and linear booster and is optimized for sparse input. It supports customized objective and evaluation functions, and performs better on several different datasets.

Random forest is a supervised classification algorithm (21). It works by learning simple decision rules extracted from the data features and overcomes the limitation of overfitting of the decision trees (22).

Logistic regression is an algorithm that classifies values through the application of a logistic function to coefficients calculated using a linear regression equation (23). It requires that the dependent variable be a second-level score or a second-level evaluation.

### Model evaluation and validation

A confusion matrix was employed to evaluate the performance of the models based on ML algorithms for the assessment of hypertension risk. The distinguishing abilities of the risk assessment model were evaluated with the receiver operator characteristic (ROC) curve and the AUC (24). The performance of the models was evaluated by calculating the sensitivity (true positive rate, TPR), specificity (true negative rate, TNR), positive predictive value (PPV), negative predictive value (NPV), accuracy (ACC), and F1-score (25, 26).

### Determination of the cut-off point

The evaluations were kinds of probabilities; thus, a cut-off point was needed to classify the prediction probabilities. The probability of having hypertension was represented by “P” in the model. The cut-off point was utilized to classify the evaluated probabilities belonging to the positive results or negative results. We adopted a cut-off point of 0.5 in this study, which meant that participants were evaluated to be at high risk of hypertension when *P* ≥ 0.5; otherwise, they were not.

### Statistical analysis

Basic descriptive statistics were used to depict the characteristics of the subjects, including demographic characteristics and health-related factors. All normally distributed measurement data are depicted as the mean ± standard deviation (X ± SD), nonnormally distributed measurement data are reported as the median (25th percentile, 75th percentile), and the counting data are expressed as the frequency and proportion. Between groups, normally distributed measurement data were compared by *T*-test, nonnormally distributed measurement data were compared by rank sum test, and the counting data were analyzed by chi-square test. *P* < 0.05 were considered statistically significant. All statistical analyses were performed using IBM SPSS Statistics version 22.0 (IBM Corp., Armonk, NY, USA).

For the assessment models, ML algorithms, XGBoost, random forest and logistic regression were utilized for the evaluation of the risk of hypertension and the effects of the risk factors. Python 3.7.3 was used for the construction of the risk assessment models of hypertension.

### Reporting guidelines

Results are presented in accordance with the Transparent reporting of a multivariable prediction model for individual prognosis or diagnosis (TRIPOD) guidelines. STROBE and RECORD guidelines for observational studies and studies using routinely collected health data were also considered. The study was conducted in accordance with relevant institutional guidelines.

## Results

### Characteristics of the study population

A total of 40,261 subjects were included, with a mean age of 72.429 ± 7.643 years, and the mean age of patients with hypertension was 73.216 ± 7.696 years. The sample prevalence of hypertension was almost 62.19%. The differences in age, diabetes status, urinary protein level, BMI, EHSA, Cr level, SBP, WC, smoking status, LDL-C level, HDL-C level, frequency of drinking, glucose level, urea nitrogen level, TC level, DBP, exercise frequency, high salt consumption, and TG level between participants with hypertension and normotensive participants were statistically significant (*P* < 0.01). There were no statistically significant differences (*P* > 0.05) in terms of time spent engaged in exercise. The characteristics of the study participants are summarized in Table 1.

### Construction of the risk assessment models

The training set and validation set were utilized to determine the optimal parameters for XGBoost, random forest and logistic regression. The parameters of each model under optimal performance are exhibited in Table 2. For other unlisted parameters in the three ML algorithms, default values were set.

**Table 2:** Configuration of parameters in each ML algorithm.

**ML algorithm**	**Parameter**	**Value range**	**Optimal value**
XGBoost	learning_rate	[0, 0.3]	0.05
	n_estimators	[100, 500]	200
	gamma	[0, 20]	5
	subsample	[0, 0.9]	0.4
	colsample_bytree	[0.5, 0.9]	0.9
	min_child_weight	(1, 6)	5
	max_depth	(2, 8)	6
	objective	-	binary:logistic
Random forest	n _estimators	[1, 50]	40
	criterion	gini	gini
	max_depth	none	none
	min_samples_split	[5, 200]	200
	min_samples_leaf	[1, 50]	1
	max_features	auto	auto
Logistic regression	C	[0, 200]	100
	class_weight	none	none
	max_iter	[10, 100]	10
	solver	-	liblinear

### Feature importance

**Figure 1:**
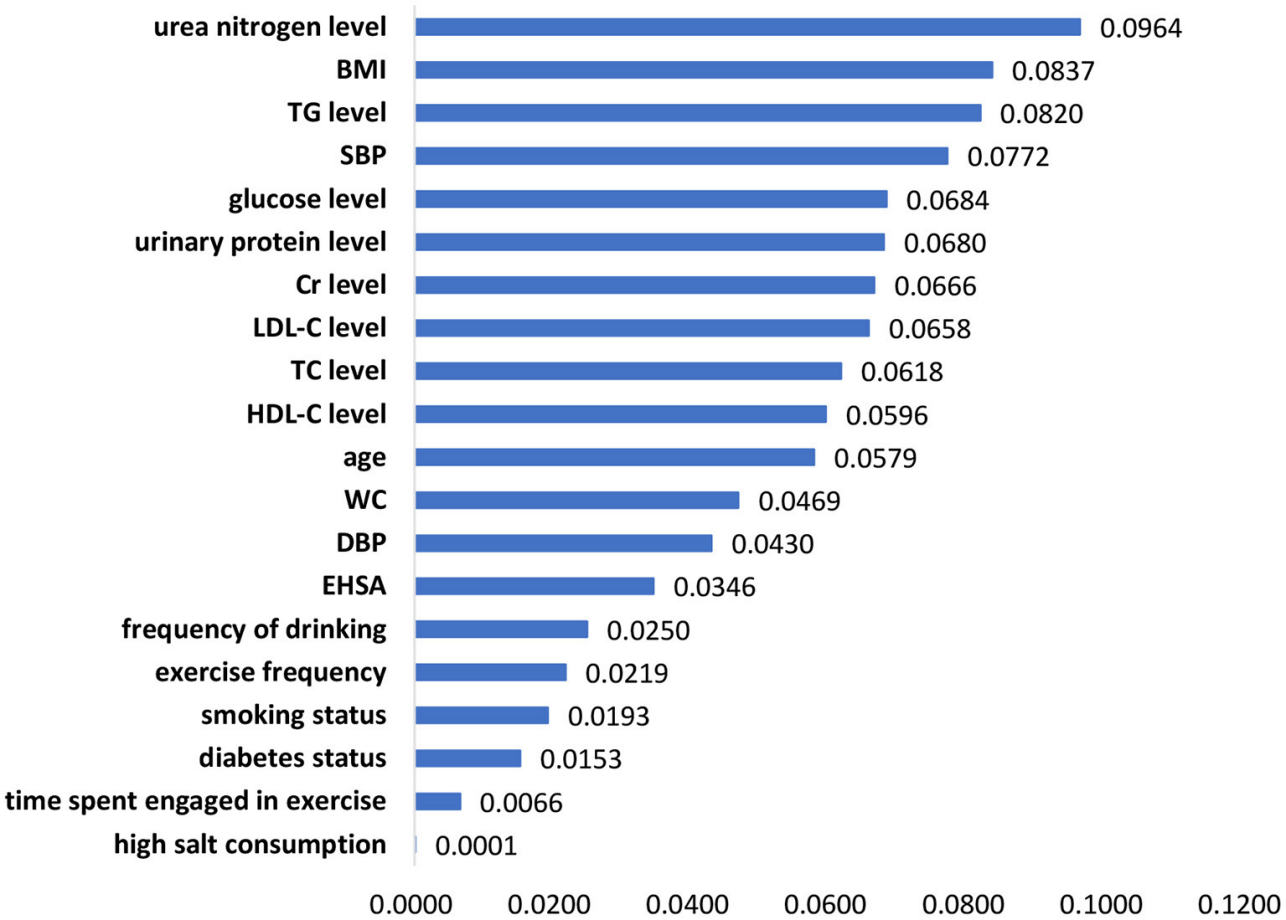
Feature importance in the XGBoost model.

**Figure 2:**
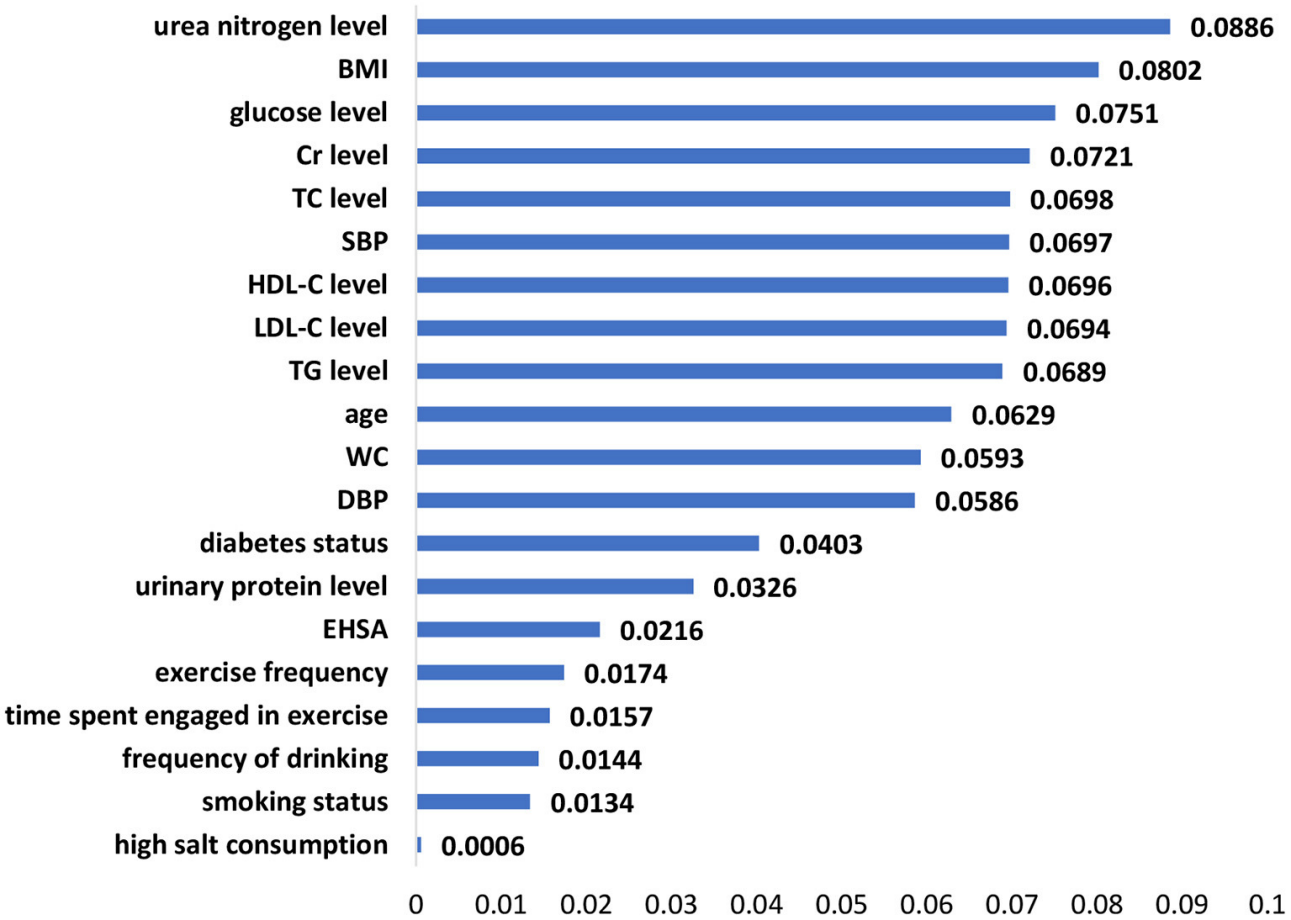
Feature importance in the random forest model.

**Figure 3:**
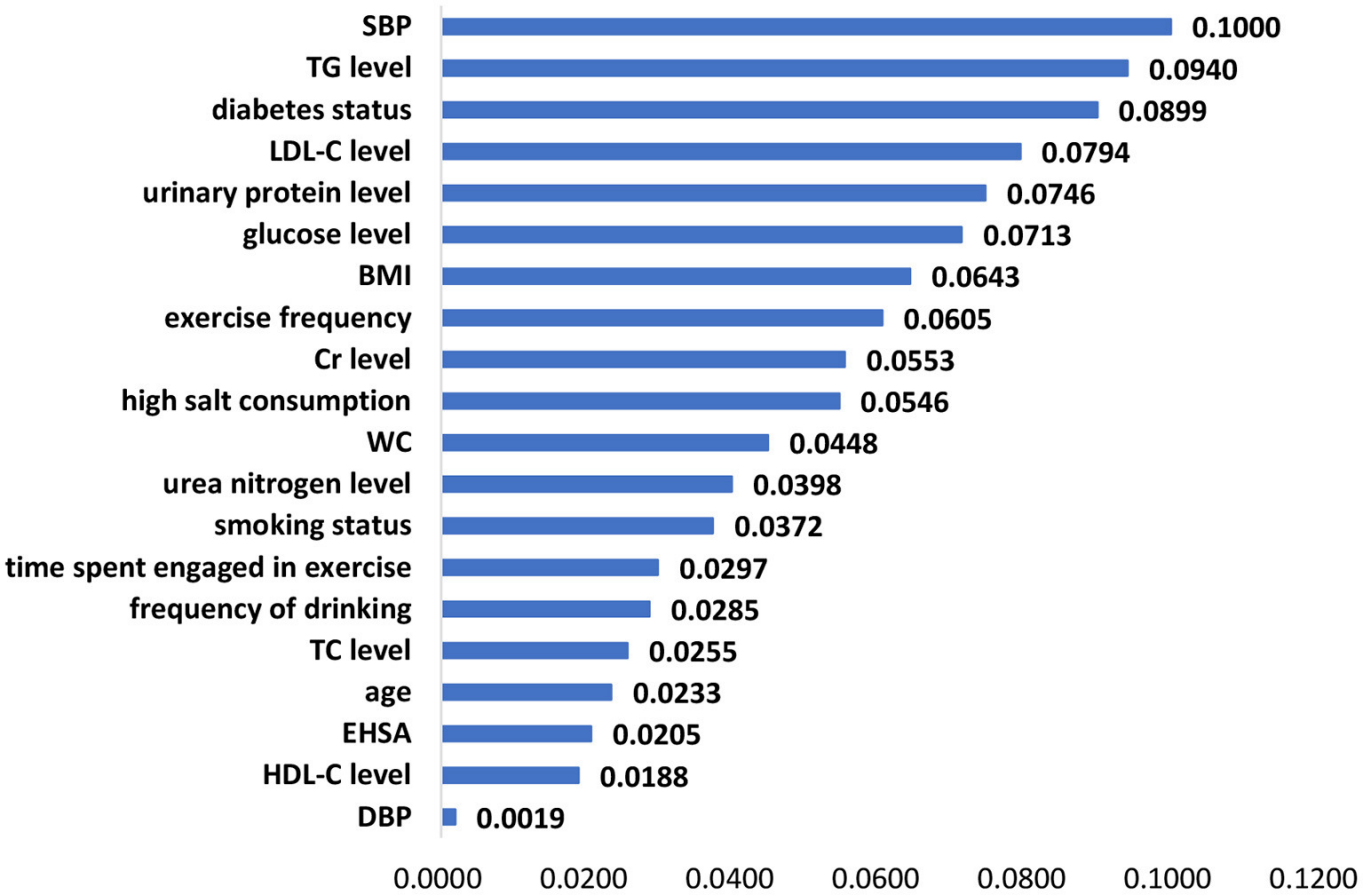
Feature importance in the logistic regression model.

The significant features of the XGBoost model, random forest model and logistic regression model are listed in Figures 1–3, respectively. The urea nitrogen level was the highest ranked feature for predicting hypertension in both the XGBoost model and the random forest model. BMI, SBP, TG level, Cr level, LDL-C level, and glucose level were ranked in the top 10 in all three models.

### Model performance

We utilized various methods and evaluation metrics to assess the performances of the XGBoost, random forest, and logistic regression models in the training, validation, and testing sets. Overall, the XGBoost model outperformed the other two models in TPR (0.864), TNR (0.488), PPV (0.735), NPV (0.686), ACC (0.722), F1-score (0.795), and AUC (0.765) in the testing set (Table 3).

**Table 3:** The fitting results for the XGBoost, random forest, and logistic regression models for the training, validation, and testing sets.

**ML algorithm**	**Dataset**	**TPR**	**TNR**	**PPV**	**NPV**	**ACC**	**F1-Score**	**AUC**
XGBoost	Training	0.886	0.530	0.756	0.739	0.752	0.816	0.818
	Validation	0.862	0.480	0.732	0.678	0.717	0.791	0.753
	Testing	0.864	0.488	0.735	0.686	0.722	0.795	0.765
Random forest	Training	0.896	0.434	0.723	0.718	0.722	0.800	0.782
	Validation	0.871	0.446	0.721	0.678	0.711	0.789	0.745
	Testing	0.816	0.548	0.748	0.644	0.714	0.780	0.756
Logistic regression	Training	0.827	0.411	0.698	0.591	0.670	0.757	0.705
	Validation	0.822	0.418	0.699	0.588	0.669	0.756	0.692
	Testing	0.829	0.430	0.705	0.604	0.678	0.762	0.707

**Figure 4:**
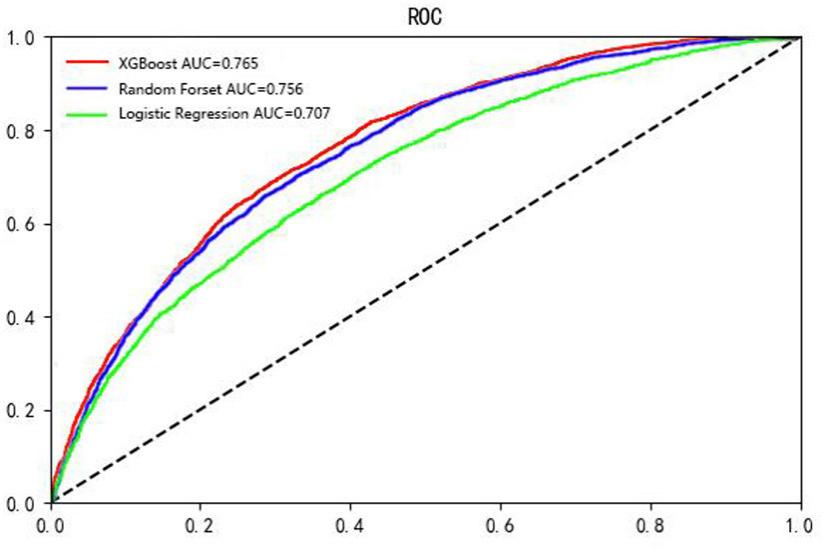
The ROC curves obtained from the XGBoost model, random forest model and logistic regression model. X axis: 1-specificity, Y axis: sensitivity. The reference line is shown as a dashed line (the black line).

Figure 4 summarizes the ROC curve areas obtained from the XGBoost model, random forest model and logistic regression model in the testing set. The areas under the ROC curves were different among the three models. The AUCs for the test set were 0.765 for XGBoost, 0.756 for random forest, and 0.707 for logistic regression (Table 4). The AUC of the XGBoost model was higher than that of the random forest and logistic regression models. Our results demonstrated that the XGBoost model had better predictive performance than the random forest and logistic regression models.

**Table 4:** AUCs for the XGBoost, random forest, and logistic regression models for the training, validation, and testing sets.

**ML algorithm**	**Dataset**	**AUC**
XGBoost	Training	0.818
	Validation	0.753
	Testing	0.765
Random forest	Training	0.782
	Validation	0.745
	Testing	0.756
Logistic regression	Training	0.705
	Validation	0.692
	Testing	0.707

## Discussion

Among the 20 selected features in this study, BMI, SBP, TG level, Cr level, LDL-C level, and glucose level had a strong effect on hypertension prediction and were included among the top 10 in the ranking of the feature importance for all three models. Similar to the results of previous studies, features such as age (27–29), BMI (28, 30), diabetes status (28), Cr level (26), blood pressure (29), WC (31), smoking status (28), LDL-C level (26, 28), HDL-C level (26), drinking (28), glucose level (32), TC level (26, 27), exercise (33), salt intake (34), and TG level (27) were identified as predictors of hypertension in the risk assessment model of hypertension.

However, to the best of our knowledge, urinary protein level, urea nitrogen level, and EHSA entered the models as new components that have not been included in risk evaluation models of hypertension in previous studies.

A study collected data from three exams in the Strong Heart Study, explored the risk factors for hypertension by means of generalized linear models and demonstrated that systolic blood pressure was significantly and positively associated with albuminuria, age, and obesity and negatively associated with smoking. Moreover, participants with more severe albuminuria status or older age developed higher SBP, while DBP was not significantly affected by the albuminuria status (35). This study in American Indians revealed that having macro/microalbuminuria is a significant risk factor for hypertension, which can explain why urinary protein level was selected as one of the features in our model to some extent. Urinary protein level may also affect the development of hypertension in Chinese individuals or facilitate the risk assessment of hypertension in Chinese individuals. Furthermore, Kim et al. reported that subjects with high normal BP had an independently significant association with microalbuminuria by performing a multiple logistic regression analysis, with an odds ratio of 1.692 and a 95% confidence interval of 1.097 to 2.611 (36). These results from a Korean population indicated that compared to individuals with normal BP, those with high normal BP have more risk factors for hypertension and cardiovascular diseases, for instance, albuminuria. Since the incidence of urinary protein was significantly higher in the prehypertensive population than in the normal population, urinary protein level should receive attention in future predictive studies and intervention measures.

Although we rarely found urea nitrogen level to be included as a predictive factor in the risk prediction models, it was found to be a significant risk factors for hypertension. A case-control study conducted among university staff found that staff with high serum urea levels had a higher risk of hypertension than those with normal urea levels (OR = 1.452), which implies that the level of urea is also very important as one of the risk factors for hypertension (37). Not coincidentally, this phenomenon has been found among middle-aged and elderly people. SBP was positively correlated with the blood urea nitrogen concentration (*r* = 0.16424, *P* = 0.0105) and the blood uric acid concentration (*r* = 0.16023, *P* = 0.0126) among middle-aged and older-aged populations in Guangzhou, China, as well as DBP (blood urea nitrogen concentration: *r* = 0.13506, *P* = 0.0358; blood uric acid concentration: *r* = 0.16562, *P* = 0.0099) (38). The results of stepwise regression analysis also indicated that there was still a significant positive correlation between SBP, DBP and concentrations of blood urea nitrogen and blood uric acid. The role of urea nitrogen level, one of the features entered into our risk assessment model, in the occurrence and development of hypertension still needs to be further investigated.

EHSA was also one of the predictors entered into our model. Kaplan and Camacho have already reported that the association between level of perceived health and mortality persisted in multiple logistic analyses controlling for age, sex, physical health status, health practices, social network participation, income, education, health relative to peers of the same age, anomy, morale, depression, and happiness (39). The results reminded us that self-assessment of health might serve as a comprehensive reflection of unmeasurable factors and as an indication of some underlying diseases or an early stage of the diseases. Evidence has shown that psychosocial factors exert strong effects on health status measures (40). Zhang et al. revealed that the proportion of elderly individuals with poor or normal health self-assessments who were suffering from common chronic diseases was significantly increased (41). The health self-assessment epitomizes the health concept and self-perception of health status of elderly individuals to some extent, which might have an underlying predictive value on the prediction of the risk of hypertension and should thus be given more attention in future research, as well as the practice in primary care.

Unlike traditional risk assessment methods, our study employed ML algorithms for model construction. XGBoost exhibited the best performance compared to random forest and logistic regression. Logistic regression assumes that every variable should be independent, and the model possesses only a linear partition surface. However, the associations between exposure factors and diseases are often affected by various confounding factors, which leads to the large deviation and low accuracy when fitting the model through logistic inference. In contrast, XGBoost and random forest are nonparametric algorithms (42) that do not assume that a functional relationship between the features and outcomes exists, as required by logistic regression models. A greedy algorithm is executed to determine the optimal splits in the data that reduce the entropy of the outcome to the utmost extent during every split. As a result, once a feature is selected, the significance of any highly related feature will decrease greatly due to the completion of the effective split done by the original feature previously. Consequently, the entropy of the outcome will no longer be reduced effectively by related features. Therefore, XGBoost and random forest are robust to related features. The reason why XGBoost outperforms the other methods may be that it introduces the regularized loss function (43) and combines gradient lifting algorithms and decision trees, which preserves the correlation between features during the modeling process (44).

In terms of performance, the XGBoost-based hypertension prediction model proposed by the Japanese group showed an AUC of 0.877 (10), while the hypertension risk assessment model proposed in this study exhibited an AUC of 0.765. The explanation for this discrepancy may be the difference in ethnic populations. According to previous studies, different ethnic populations have different characteristics of hypertension, which may affect the discrepancies in the AUCs for different models (45, 46). Meanwhile, the difference between age range of the subjects may also contribute to the discrepancy in the model performance. For instance, in a study regarding assessing the relationship between nerves and cancer using machine learning methods, the authors found that the performance of the model trained on the young dataset was much better than that trained on the elderly dataset and the whole age dataset, and the performance of the model trained on the whole age dataset was slightly better or similar to that trained on the elderly dataset (47). The findings from these studies suggested that we should further investigate the effect of the difference in subjects' age range on the performance of hypertension models in the future. Compared with other models used to predict hypertension (11), the results from the proposed XGBoost prediction model in the present study did not show a higher AUC. The variable selection may partially explain the discrepancy.

After the risk assessment of hypertension, subsequent interventions and management to prevent or postpone the occurrence and development of hypertension are crucially important in high-risk populations. Continuous monitoring and management are imperative for high-risk patients. On the one hand, realtimeness and continuity monitoring can detect any problem without delay. On the other hand, early signs of detected symptoms can alert both general practitioners (GPs) and individuals in a timely manner. For high-risk populations, corresponding individual intervention strategies targeting the main risk factors should be prescribed by GPs in primary care. For instance, lifestyle factors such as exercise, eating habits, and drinking habits can be improved under the guidance of GPs after risk assessment. Evidence has revealed that a high concentration of parks or playgrounds in residential areas may reduce the risk of hypertension, which is mainly attributable to the cultivation and formation of exercise habits and implies the importance of interventions in communities (48).

However, there were several limitations in our study. One of the limitations of the study was that it had a cross-sectional design, and the results could not indicate causality in this situation. A prospective cohort study is needed to further identify the cause-and-effect relationships. Second, the risk assessment model was designed considering only variables available in the setting of primary care, and variables regarding mental health and hereditary factors were not included. Third, we measured several variables, such as age, urinary protein level, BMI, and Cr level, on only a single occasion and did not take changes in these variables into consideration.

In conclusion, XGBoost outperformed random forest and logistic regression models in predicting the risk of hypertension in primary care settings. Early identification and the corresponding preventive strategies in primary care remain insufficient in China. Integration of such a risk assessment model into primary care may help general practitioners target populations at high-risk for hypertension, tailor the corresponding preventive measures and treatment strategies to those at high risk, improve the awareness of residents regarding health risks and their adherence toward targeted intervention, and eventually facilitate individuals' health and quality of life while decreasing healthcare costs.

## Data availability statement

The original contributions presented in the study are included in the article/Supplementary material, further inquiries can be directed to the corresponding authors.

## Ethics statement

The studies involving human participants were reviewed and approved by the Ethics Committees of Tongji University. The patients/participants provided their written informed consent to participate in this study.

## Author contributions

NC was involved in designing the study, analyzing the results, and wrote the manuscript. FF performed data collection, proofread the manuscript, and modified the format. DY, ZW, and JS supervised the work and were involved in the study design. JG and YY helped with data interpretation and graphing. YG, HJ, and QC revised the manuscript. All authors reviewed and approved the final version of the manuscript.

## Funding

This study was supported by grants from Soft Science Project of the Shanghai Science and Technology Commission (22692107200), Shanghai Education Science Research Project (C2021039), the Natural Science Foundation of China (71774116 and 71603182), Shanghai Public Health Outstanding Young Personnel Training Program (GWV-10.2-XD07), National Key Research and Development Program of China (2018YFC2000700, SQ2022YFC3600172), and Shanghai Pujiang Program (2020PJC080). The funding agencies had no role in the design of this study nor any role during its execution, analyses, data interpretation, or decision to submit results.

## Conflict of interest

The authors declare that the research was conducted in the absence of any commercial or financial relationships that could be construed as a potential conflict of interest.

## Publisher's note

All claims expressed in this article are solely those of the authors and do not necessarily represent those of their affiliated organizations, or those of the publisher, the editors and the reviewers. Any product that may be evaluated in this article, or claim that may be made by its manufacturer, is not guaranteed or endorsed by the publisher.
